# Supplementary material for: Resolving the spin splitting in the conduction band of monolayer MoS2
Source: Nat Commun. 2017 Dec 5;8:1938. doi: 10.1038/s41467-017-02047-5 (PMC5717150; doi:10.1038/s41467-017-02047-5)
Supplement: Supplementary file 1 — Supplementary Information [file 41467_2017_2047_MOESM1_ESM.pdf]

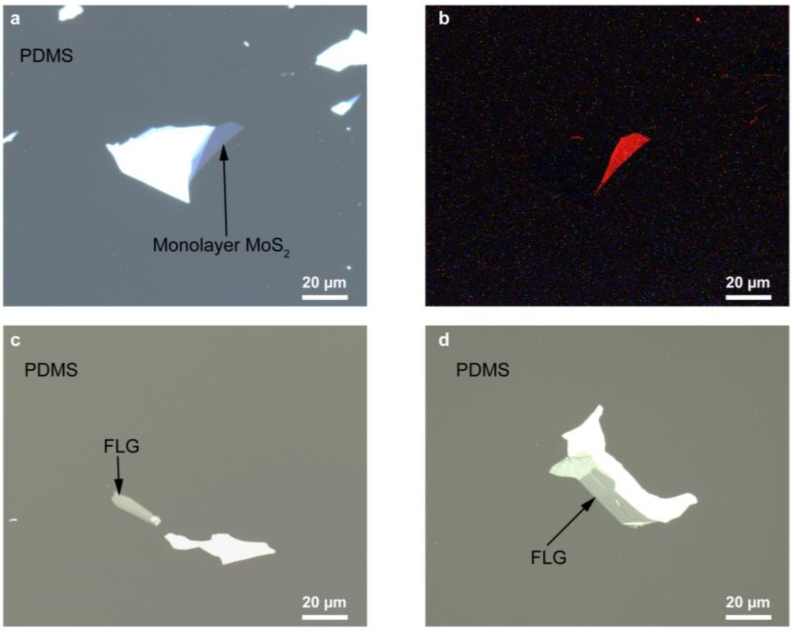


**Supplementary Figure 1**. **Exfoliated $MoS_2$ and FLG crystals**. **a,** Optical micrograph of the exfoliated monolayer $MoS_2$ on PDMS substrate. **b,** Dark-field microscope PL of the crystal in **a**. There is a strong optical signal only from monolayer $MoS_2$. **c and d,** Optical micrographs of the FLG flakes used as source and drain contacts to the monolayer $MoS_2$.

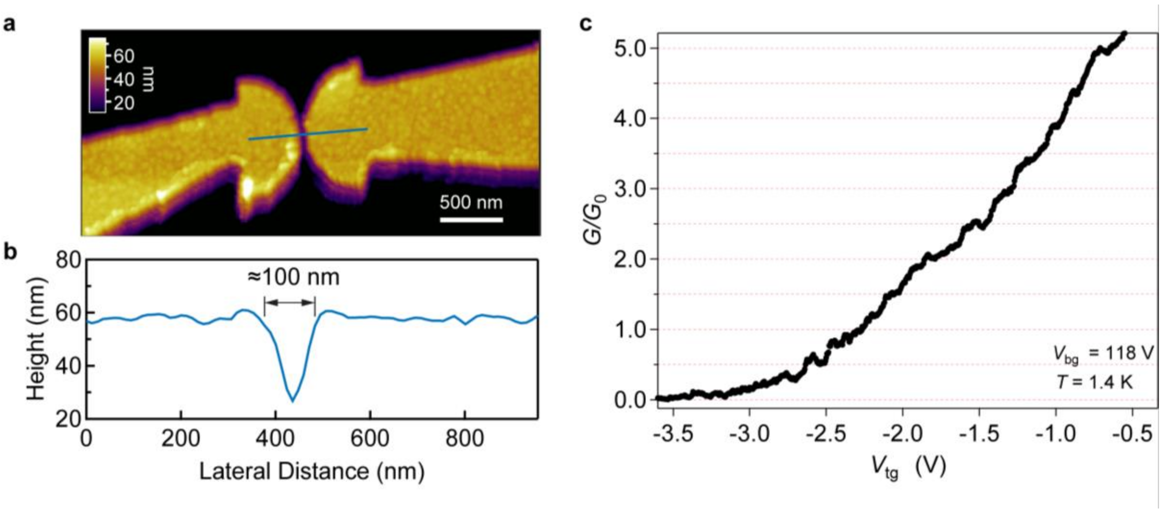


**Supplementary Figure 2. Observation of conductance quantization in another similar device. a,** AFM image of the split gate geometry, which is very similar to that of the device in the main text. **b**, Height profile along the blue line displayed in **a**. The distance between the two top gates is about 100 nm. **c,** Conductance of the device as function of the applied top-gate voltage after subtraction of series resistance of 8.3 kΩ. Clear quantization steps in whole and half values of $G_0 = 2e^2/h$ are observed.

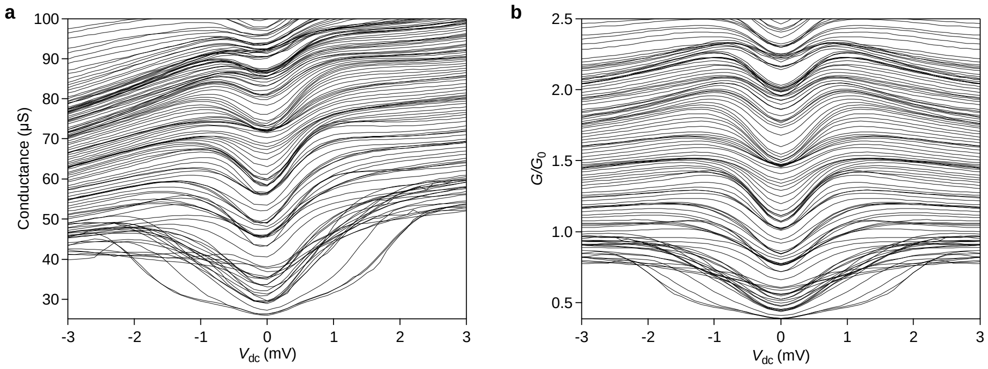


**Supplementary Figure 3. Correction of the self-gating effect and series resistance. a,** Raw data of the map at B = 0 T. The asymmetry originates from the self-gating effect. **b**, The same map as in **a** after averaging for positive and negative bias and subtraction of series resistance.

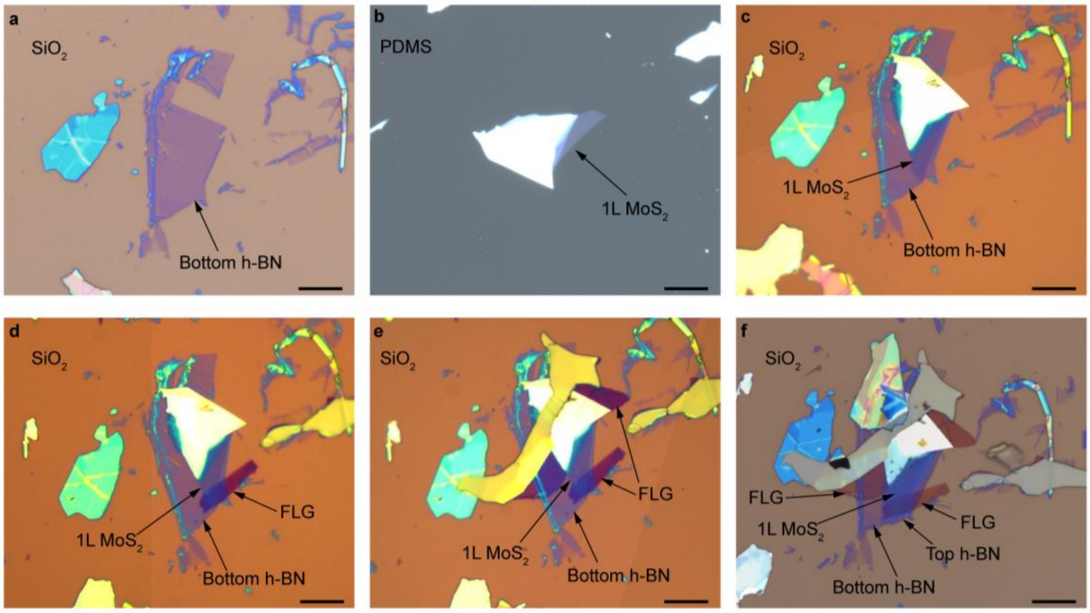


**Supplementary Figure 4. Transfer sequence of the heterostructure device. a,** Optical image of the exfoliated ~5 nm thick h-BN crystal serving as a bottom substrate later on. **b**, Optical image of the monolayer $MoS_2$ exfoliated on PDMS used in this work. **c,** Optical micrograph showing the 1L $MoS_2$ crystal after its deposition on h-BN from **a**. **d,** Image of the heterostructure after transfer of the first few-layer graphene crystal form PDMS to the substrate. **e,** Optical image of the heterostructure after deposition of the second FLG crystal. **f,** Final completed 1L $MoS_2$ encapsulation after deposition of the top h-BN crystal.

### Supplementary Note 1: Material preparation

All crystals needed for the realization of a heterostructure were exfoliated on a PDMS substrate by means of mechanical cleavage. Thin crystallites were detected optically. In Supplementary Figure 1a we show the single layer $MoS_2$ used in this work after exfoliation on PDMS. For further confirmation, we perform dark field microcopy photoluminescence measurements[1] (Supplementary Figure 1b). In this optical picture we see clearly the very strong homogeneous emission from the monolayer, while thicker $MoS_2$ crystallites remain dark, because their bandgap is indirect. As shown previously, very good ohmic contact to $MoS_2$ can be realized with few layer graphene (FLG).[2] For this, we exfoliate NGS graphite on PDMS substrates and select crystals that are thin and large enough to serve later as source and drain electrodes (Supplementary Figure 1c and d).

### Supplementary Note 2: Conductance quantization in an identical device

For further confirmation of the observed conductance quantization, we prepared in identical manner another device. It consists of a monolayer $MoS_2$ contacted with few-layer graphene electrodes and encapsulated between two h-BN layers. The geometry of the deposited split top-gates is very similar to the device presented in the main text. An AFM image is presented in Supplementary Figure 2a. Following the blue line (Supplementary Figure 2b) we estimate the separation between the gates to be about 100 nm. Further, we study the conductance of this device at base temperature of 1.4 K and at high doping as function of the applied top-gate voltage. The back-gate in this case is kept at constant 118 V and the top gate voltage is swept from 0 to -3.6 V (Supplementary Figure 2c).

At $V_{tg}$ = -0.8 V we can observe the first plateau in the conductance curve. When the top-gate voltage is further reduced, we see series of ten conductance steps separated by steep transitions down to $0.5\times G_0$. At voltages below -3 V the $MoS_2$ channel is completely pinched-off. With this, we confirm the appearance of the same conductance quantization in whole and half values of $2e^2/h$ in another device, which endorse our observation that in a monolayer $MoS_2$ QPC all state degeneracies are lifted.

### Supplementary Note 3: Self-gating effect

Supplementary Figure 3a shows the raw data acquired for the differential conductance of the sample while sweeping the dc offset at a constant top-gate voltage. There is a clear asymmetry in the map. At negative applied dc bias voltages, the conductance decreases, while at positive biases it increases. We assume that this is due to the self-gating effect, which was also observed in other QPC devices.[3,4] Note that unlike other reports, the contact material of our device is a semimetal (FLG) and also prone to gating. In order to correct for this effect, we average between the values of conductance obtained at positive and negative biases:[3]

$$G^*(V_{\mathrm{dc}}) = \frac{1}{2}\left[G(+V_{\mathrm{dc}}) + G(-V_{\mathrm{dc}})\right]$$

The resulting symmetric corrected map in this case for $B$ = 0 T is presented in Supplementary Figure 3b. The characteristic dip of conductance around $V_{dc}$ = 0 mV is still pronounced, as it is least effected by the gating.

## Supplementary Note 4: Transfer procedure

The heterostructure was assembled by sequential deposition of exfoliated crystals from a PDMS on a $SiO_2$ substrate following the recipe of Castellanos-Gomez et. al.[5] We start with an approximately 5 nm thick h-BN crystal exfoliated on a Si/$SiO_2$ substrate (Supplementary Figure 4a). Then, we select a 1L $MoS_2$ crystal exfoliated on PDMS substrate to be deposited on the h-BN crystal (Supplementary Figure 4b). We align the PDMS stamp with the crystal facing down above the target substrate and approach slowly. When the stamp and the crystals are in contact, we heat up the substrate up to 50 °C in order to improve the adhesion between $MoS_2$ and h-BN. After waiting for about 5 minutes, we slowly raise the stamp. The $MoS_2$ crystal is deposited on the target substrate (Supplementary Figure 4c). Following this procedure, we deposit two few layer graphene crystals (Supplementary Figure 4d and e) and finally the top h-BN crystal (Supplementary Figure 4f) in order to completely encapsulate the single layer $MoS_2$. The heterostructure produced in this way is then annealed in high vacuum at 360°C for 8 hours in order to improve adhesion between the layers and remove trapped impurities and water between them.

## Supplementary References


1. Alexeev, E. M. *et al.* Imaging of Interlayer Coupling in van der Waals Heterostructures Using a Bright-Field Optical Microscope. *Nano Lett.* **17,** 5342–5349 (2017).
2. Cui, X. *et al.* Multi-terminal transport measurements of $MoS_2$ using a van der Waals heterostructure device platform. *Nat. Nanotechnol.* **10,** 534–540 (2015).
3. Kristensen, A. *et al.* Bias and temperature dependence of the 0.7 conductance anomaly in quantum point contacts. *Phys. Rev. B* **62,** 10950–10957 (2000).
4. Scappucci, G. *et al.* Conductance quantization in etched Si/SiGe quantum point contacts. *Phys. Rev. B* **74,** 035321 (2006).
5. Castellanos-Gomez, A. *et al.* Deterministic transfer of two-dimensional materials by all-dry viscoelastic stamping. *2D Mater.* **1,** 011002 (2014).
